# Modeling Mesothelioma Risk Associated with Environmental Asbestos Exposure

**DOI:** 10.1289/ehp.9900

**Published:** 2007-03-22

**Authors:** Milena Maria Maule, Corrado Magnani, Paola Dalmasso, Dario Mirabelli, Franco Merletti, Annibale Biggeri

**Affiliations:** 1 Cancer Epidemiology Unit, CeRMS and CPO Piemonte, University of Turin, Turin, Italy; 2 Unit of Medical Statistics and Epidemiology, Department of Medical Sciences, University of Eastern Piedmont and CPO Piemonte, Novara, Italy; 3 Interdepartmental Center ‘G. Scansetti’ for the Study of Asbestos and other Toxic Particulates, University of Turin, Turin, Italy; 4 Medical Statistics Unit, Department of Public Health and Microbiology, University of Turin, Turin, Italy; 5 Department of Statistics ‘G. Parenti’, University of Florence, Florence, Italy; Department of Statistics, Biostatistics Unit, Institute for Cancer Prevention (CSPO), Florence, Italy

**Keywords:** asbestos, mesothelioma, spatial models

## Abstract

**Background:**

Environmental asbestos pollution can cause malignant mesothelioma, but few studies have involved dose–response analyses with detailed information on occupational, domestic, and environmental exposures.

**Objectives:**

In the present study, we examined the spatial variation of mesothelioma risk in an area with high levels of asbestos pollution from an industrial plant, adjusting for occupational and domestic exposures.

**Methods:**

This population-based case–control study included 103 incident cases of mesothelioma and 272 controls in 1987–1993 in the area around Casale Monferrato, Italy, where an important asbestos cement plant had been active for decades. Information collected included lifelong occupational and residential histories. Mesothelioma risk was estimated through logistic regression and a mixed additive–multiplicative model in which an additive scale was assumed for the risk associated with both residential distance from the plant and occupational exposures. The adjusted excess risk gradient by residential distance was modeled as an exponential decay with a threshold.

**Results:**

Residents at the location of the asbestos cement factory had a relative risk for mesothelioma of 10.5 [95% confidence interval (CI), 3.8–50.1), adjusted for occupational and domestic exposures. Risk decreased rapidly with increasing distance from the factory, but at 10-km the risk was still 60% of its value at the source. The relative risk for occupational exposure was 6.0 (95% CI, 2.9–13.0), but this increased to 27.5 (95% CI, 7.8–153.4) when adjusted for residential distance.

**Conclusions:**

This study provides strong evidence that asbestos pollution from an industrial source greatly increases mesothelioma risk. Furthermore, relative risks from occupational exposure were underestimated and were markedly increased when adjusted for residential distance.

Asbestos is the only established causal factor for pleural and peritoneal malignant mesotheliomas (MM) and one of the main occupational risk factors for lung cancer (International Agency for Research on Cancer 1987). Reports from many countries have described cases of mesothelioma related to occupational exposure to various types of asbestos (McDonald and McDonald 1996), although not all cases could be ascribed to occupation. In the 1980s Enterline (1983) hypothesized that one-third of all mesotheliomas in the United States could have been due to nonoccupational exposure. These cases could be linked to domestic and neighborhood exposures to asbestos (Gardner and Saracci 1989; Hansen et al. 1998; Hillerdal 1999; Magnani et al. 2000, 2001) or environmental exposure to naturally occurring asbestos or asbestiform fibers (Baris et al. 1987; Luce et al. 2000; Mirabelli and Cadum 2002; Pan et al. 2005; Paoletti et al. 2000; Sakellariou et al. 1996). A meta-analysis by Bourdes et al. (2000), based on studies available in the early 1990s, estimated relative risks of pleural mesothelioma ranging between 4.0 and 23.7 for household exposure and between 5.1 and 9.3 for neighborhood exposure.

A number of studies have investigated the health effects of exposures to environmental asbestos from industrial or natural asbestos sources. Newhouse and Thompson (1993) found an increased MM risk for people living within 800 m of an asbestos factory. Schneider et al. (1996) observed an association of MM with neighborhood asbestos exposure for residents outside Hamburg, Germany. Hansen et al. (1998) were the first to estimate the quantitative exposure–response relationship between MM and environmental asbestos exposure among residents living near a crocidolite mine in Wittenoom, Australia. Pan et al. (2005) have recently calculated that the odds of mesothelioma decreased approximately 6.3% with every 10 km of distance from the nearest source of naturally occurring asbestos. Their study was register based and included nearly 3,000 cases. Although no residential histories were available for the subjects of the study, the proxy used (residence at the time of diagnosis, which does not consider the long latency of mesothelioma) was precisely geocoded, as well as the location of the ultra-mafic rocks acting as the principal source of naturally occurring asbestos. Other studies found negative or inconclusive results (Hammond et al. 1979; McDonald and McDonald 1980; Teta et al. 1983).

The risk of mesothelioma from nonoccupational asbestos exposure is unquestionably important from a public health point of view and is crucial in the investigation of the exposure–response relationship, which cannot be exclusively inferred from studies on workers, typically adult, male, and subjected to very high fiber concentrations (Goldberg and Luce 2005).

In Italy several studies concerning mesothelioma risk in asbestos cement (AC) workers (Magnani et al. 1996), their wives (Magnani et al. 1993), and the general population (Magnani et al. 1995) have been conducted in Casale Monferrato, where the largest Italian AC plant was active for many decades. Incidence rates for the general population were estimated to be as much as 10 times that in other industrial areas of Northern Italy (Magnani et al. 1995). The Casale case series was also included in an international multicentric case–control study that was the first to estimate a nonoccupational risk for mesothelioma (Magnani et al. 2000) and found an odds ratio (OR) of 11.5 [95% confidence interval (CI), 3.5–38.2] for high probability of environmental exposure. The most recent study conducted in Casale (Magnani et al. 2001) showed that environmental exposure caused a greater risk than domestic exposure, and concluded that there was a suggestion of a spatial trend with increasing distance. However, some important issues remained unresolved. In particular, the most recent analysis (Magnani et al. 2001) measured the distance from the factory only for the dwellings located within the town boundaries, and it was not possible to estimate mesothelioma risk for greater distances. Furthermore, the functional form of the dependence of risk on the distance between residence and the factory was unknown.

Our present study addresses the questions above by gathering new information on residential histories of the subjects and through a detailed analysis of spatial variation of MM risk in the area of Casale Monferrato.

## Materials and Methods

### The factory and the town

The AC plant in Casale Monferrato had been active from 1907 to 1985, producing plane and corrugated sheets, pipes and pressure pipes, and special AC products. The size of the work force varied over time and never exceeded 1,500 workers. In 1981 the company reported using 15,000 tons of asbestos (10% crocidolite) (Magnani et al. 1996). Processes gave origin mostly to diffuse emissions from building openings such as shed windows. Local exhausts were not installed until 1978 and were limited to the lathes used for machining pressure pipes. Only the power plant had a stack, which entailed no emission of asbestos fibers (AF). The factory is upwind from the town—about 1,500 m from the center and 250 m from the closest residential areas. As far as we can infer from its raw materials and products, airborne emissions from the AC plant included both chrysotile and crocidolite fibers, and the same should apply to waste-waters and waste materials. Environmental asbestos concentration was measured shortly before the factory shutdown (1985) or afterward but never during the 1950s and 1960s when the plant was fully active and when, considering mesothelioma latency, the most relevant exposures presumably occurred. Estimates reported here are the average of repeated measurements and, if not otherwise specified, they refer to airborne AF with length > 5 μm and diameter > 0.3 μm.

Marconi et al. (1989) reported asbestos concentrations ranging from 11 AF/L close to the plant (around 400 m), to 4.5 AF/L in the city center and to 1 AF/L in the city area farthest away in 1985; at that time production had already been significantly reduced. Short-term (4–8 hr) air sampling was employed. Scanning electron microscopy (SEM) was used for fiber counting (detection limit: 0.4 F/L) and energy dispersive X-ray analysis (EDXA) for fiber identification, according to the Asbestos International Association (AIA) method (AIA 1984).

The Local Health Authority (LHA) measured annual average concentrations < 1 AF/L in 1990–1991, with 12% of samples exceeding 1 AF/L (Unità Sanitaria Locale di Casale Monferrato, unpublished data). Short-term (4–6 hr) air samples were taken. Fiber counts (detection limit, 0.3 F/L) were conducted by SEM, and fibers were identified as asbestos fibers by EDXA. Sampling and analytical AIA methods were followed, but only fibers with diameter > 0.5 μm were counted.

In 1991 asbestos fibers concentrations from 2.2 to 7.4 AF/L were reported in the residential areas of Casale. The average concentration of total (any length) asbestos fibers was 48.4 AF/L (1.5 AF/L total amphiboles), versus, respectively, 0.2–12.1 and 0.0–0.2 AF/L in other industrial cities (Chiappino et al. 1991, 1993). Very long sampling times (3–7 days) were used. Fibers were counted on transmission electron microscope (TEM, detection limit not provided) and were identified by EDXA. Fibers were suspended in solvent after ashing the original cellulose acetate filter (or a portion of it), with ultrasound agitation and eventually refiltered on a nucleo-pore membrane for “indirect” TEM preparation. This technique clearly differs from the AIA method. Marconi et al. (1989) stated that calcium-rich amphiboles accounted for 15–30% of all asbestos fibers. Chiappino et al. (1991) reported that amphiboles represented almost 50% of asbestos fibers > 5 μm. In the LHA report no asbestos type–specific figure was given.

A survey on lung fiber burden in a series of consecutive necropsies supports the hypothesis that important pollution from amphiboles was present in Casale (Magnani et al. 1998). This notion is obviously relevant for our study, as the carcinogenic potency of amphiboles for inducing MM is considered much higher than that of chrysotile (Hodgson and Darnton 2000).

### Study design

This is a population-based case–control study that includes cases of pleural MM newly diagnosed between 1 January 1987 and 30 June 1993 among residents in Casale Monferrato and the surrounding area, comprising roughly 52 towns and over 100,000 inhabitants (of whom 40,000 are in Casale).

Cases were retrospectively identified through surveys of the pathology units of the hospitals serving the study area and were all histologically confirmed. Of the 123 cases included in the Piedmont Registry of Malignant Mesotheliomas files for 1987–1993 among residents in the study area, 116 (94.3%) were eligible for this study (Ivaldi et al. 1999). Controls were selected randomly either from the files of residents in the LHA or from the mortality files of residents in the same area and individually matched to cases by sex, birth date (± 18 month), vital status, and date of death (± 6 months). In subsequent analyses, individual matching was disregraded because in our study, matching variables were spatially neutral (Cuzick and Edwards 1990; Diggle 2003).

Live subjects and the closest relative of deceased subjects were interviewed from 1993 to 1995 using a standardized questionnaire, with sections designed to reconstruct the lifelong occupational history of the subjects, their spouses, relatives and any other cohabitants, and others on demographic characteristics, smoking, radiation treatment, and schools attended.

In addition to environmental exposure, represented by the distance between residence and the factory, three other main sources of asbestos exposure were identified: *a*) occupational exposure in the AC industry; *b*) domestic exposure, with which we refer to either the indoor presence of asbestos materials such as asbestos fabrics of ironing tables, fire-proof sheets for stoves and ovens, or AC materials and roofings in very close proximity to the house (e.g., garden, courtyard); and *c*) occupation in the AC industry of relatives and cohabitants. These variables were coded as dicotomic (yes/no) for all subjects. Occupational exposure in the AC industry was chosen as a proxy to asbestos occupational exposure *tout court* because it corresponds to high intensity of exposure and is highly specific. In addition to AC production and related activities (warehousing and transportation of raw asbestos and final products), no other noticeable source of asbestos exposure of industrial origin was recorded in Casale (Magnani et al. 1991). AC products were used in Casale Monferrato as elsewhere in the building industry, namely, for roofs and water pipings. There is anecdotical evidence of small quantities of asbestos having been used in a factory producing printing machines in the gaskets for the hoods of the drying sections and in a textile (silk) workshop in pipe laggings. Such exposure circumstances were actually less common than in most industrial settings in our study region. Therefore, confounding due to residual occupational exposure is unlikely. More details on data collection procedures and exposure coding have been published elsewhere (Magnani et al. 2001).

Because the focus of our present study was on the risk associated with residential distance from AC plant, special care was dedicated to the questionnaire’s section designed to reconstruct the complete residential history of all subjects, comprising all the addresses of subjects (within and outside Casale), and a description of each dwelling and its neighborhood environment. To have a comprehensive estimate of asbestos domestic exposure, we collected information on the presence and use of asbestos materials in the house or its proximities. All residential addresses obtained from the original questionnaires were compared with and completed by information from the town office registries, and coded as Universal Transverse Mercator (UTM) geographic coordinates using a global positioning system (GPS) receiver. The geographic coordinates of the AC factory location were determined in a similar manner. Because each subject had inhabited more than one dwelling, the address of the longest-held residence was chosen as a proxy to residential distance exposure, after exclusion of dwellings occupied in the last 20 years before the date of diagnosis for cases or before the date of the interview or the date of death for alive and deceased controls, respectively.

Informed consent was obtained before the study from live subjects or from the next-of-kin of deceased subjects. The study was approved by the institutional review board of the department of Biomedical Sciences and Human Oncology of the University of Turin.

### Statistical methods

Basic data analyses used logistic regression (Breslow and Day 1980) and provided risk estimates in form of ORs, adjusted for age and sex, with 95% confidence intervals (CIs).

Kernel density estimation was used to estimate and map spatial variation in disease risk (Bithell 1990; Kelsall and Diggle 1995). It was assumed that cases and controls formed two independent point-Poisson processes of different intensities. Kernel smoothing was used to estimate the density surfaces of cases and controls separately, and the risk surface was then obtained as the ratio of the two.

To analyze specifically the effects of residential distance from the AC plant on MM risk, we first defined a logistic regression model in which distance was subdivided in classes and included as a categorical variable. Furthermore, we defined a mixed additive-multiplicative model in which the odds of disease are as follows:


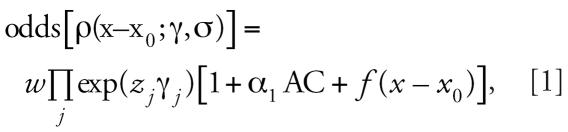


where *w* is a proportionality factor; γ*_j_* is the log OR for the *j*th multiplicative risk factor, *z_j_* ; α_1_ is the excess risk for AC occupational exposure (AC); *f*(·) is the distance excess risk function; and *d* = *x*–*x*_0_ is the distance (meters) between individual residence location and the source. The distance excess risk function was specified as:


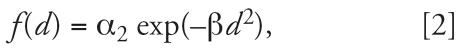


known as an exponential-threshold model (Waller and Pocquette 1999), where the parameter α_2_ represents the excess relative risk at the source and the parameter β models the exponential risk decay (per unit squared-distances). Multiplicative risk factors included age, sex, indoor or outdoor domestic exposure to asbestos, and occupation in the AC industry of any relative. An additive scale was assumed for the risk associated with residential distance and AC occupational exposure. The rationale of this choice is to ensure that the risk is unchanged at and infinite distance from the source *x*_0_. Furthermore, it is reasonable, on biological grounds, to assume that different exposures to the same agent interact additively (Pira et al. 2005).

Spatial clustering was investigated through nonparametric methods including the Cuzick–Edwards test (Cuzick and Edwards 1990) and second-order distance methods based on Ripley’s *K* and Diggle’s *D* functions (Diggle and Chetwynd 1991; Ripley 1976).

## Results

Of 116 cases and 330 controls eligible for the study, 103 (89%) and 272 (82%) agreed to participate in the study and were interviewed. There were no significant differences in age, sex, or residence between respondents and nonrespondents.

Table 1 describes the complete data set of 375 interviewed subjects. Cases and controls presented similar distributions by age, sex and vital status. Age and sex were nevertheless always included in the logistic models as potential confounders.

**Figure 1:**
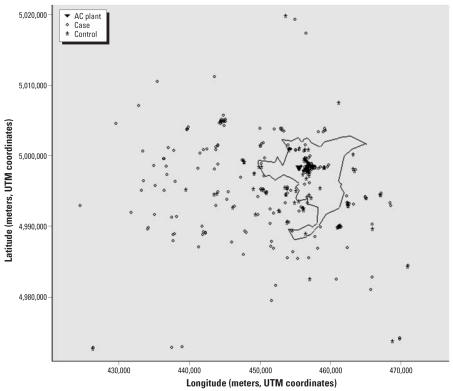
Spatial distribution of the residences of both occupationally and nonoccupationally exposed cases and controls in a geographic area of approximately 50 km^2^ around Casale. Residences are the longest-held among all residences of each individual after excluding 20 years before the date of the diagnosis of the index case. All 97 cases and 233 of 250 controls are included (17 control residences are outside the figure area). The location of the AC plant in the town of Casale is also indicated.

Geographic coordinates of the longest-held residence of each subject up to 20 years before the date of diagnosis (cases) or of the interview/death (controls) were available for 97 (94%) cases and 250 (92%) controls. The distribution of index residences in a geographic area of roughly 50 km^2^ around Casale is shown in Figure 1. Seventeen controls were outside the plot area. Longest-held addresses could be considerable distances from Casale, as subjects were only required to reside in the LHA of Casale at the moment of diagnosis. The spatial distribution of the controls on the map represents the population density in the region.

**Figure 2:**
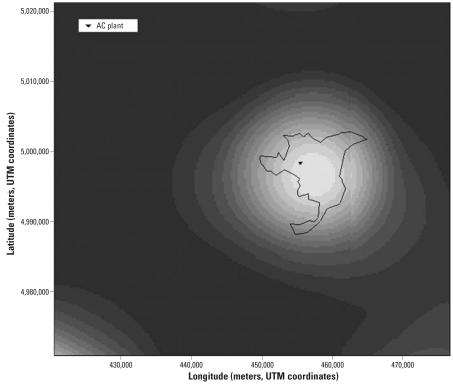
Contour plot of the ratio of case to control kernel density surfaces including both occupationally and nonoccupationally exposed individuals. Smoothing parameters were 10 km for cases and 20 km for controls. The town of Casale and the location of the AC plant are indicated.

Figure 2 shows the contour plot of the risk for MM in the same geographic area. The risk surface was estimated as the ratio of case to control kernel density surfaces, including all individuals independent of their status of occupational exposure. Risk shows a well-defined peak in the center of Casale, southeast from the AC factory, and seems to decrease monotonically in all directions.

The effects on MM risk of the three main sources of asbestos exposure that are different from residential distance exposure are shown in Table 2. Twenty-eight cases and 14 controls had worked in the AC industry, resulting in an OR of 7.1 (95% CI, 3.5–14.3) for occupational exposure. Between domestic exposure and occupational exposure of relatives, the latter was the most important, although risk estimate decreased (maintaining statistical significance) after adjusting for AC occupation.

To analyze the residential distance exposure effects, we classified individuals as resident in geographic bands at increasing distance from the AC plant (Tables 3 and 4). Table 3 shows that risk decreases with increasing distance, with strong evidence of a spatial trend (*p* < 0.0001). The band at 3–5 km from the AC factory included a remarkably high concentration of cases. Restricting the analysis to the nonoccupationally exposed subjects (Table 4) did not change risk estimates significantly. The robustness of risk estimates associated with residential distance allowed us to include all subjects in the subsequent analyses rather than restricting them to the nonoccupationally exposed subgroup, thereby improving the precision of the estimates.

Table 5 shows the relative risk for MM associated to AC occupation, domestic exposure, and relatives’ AC occupation, as estimated by the mixed additive–multiplicative model for excess relative risk, with and without adjustment for the distance of the residence from the AC plant.

**Figure 3:**
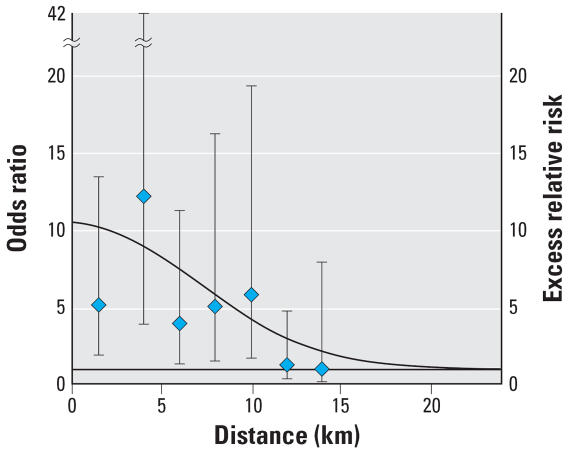
Risk of malignant mesothelioma of the pleura in Casale in relation to the distance of individuals’ longest-held residence (after exclusion of 20 years from the date of the diagnosis) from the AC plant. Risk estimates are adjusted for age, sex, occupation in the AC industry, indoor or outdoor domestic exposure to asbestos (asbestos materials in the garden, courtyard, roof, inside the house), occupation in the AC industry of any relative. ORs are estimated through the logistic model and are represented with error bars (95% CI); relative risks are estimated through the model with exponential decay with threshold of the risk by distance from the AC plant and are represented as a smooth line.

Estimates of the parameters of the mixed additive-multiplicative model (Equation 1) were α_2_ = 9.5 (95% CI, 2.8–49.1), which represents residential distance excess risk at the source, namely, a relative risk of 10.5 for hypothetical residents living at the AC plant (at zero distance from the source); and β = 0.11 × 10^−7^ (95% CI, 0.48 × 10^−8^ to 0.25 × 10^−7^), which represents risk decay rate per unit squared-distances (distance measured in meters) moving away from the source. At 10 km from the source, the relative risk was estimated to have decreased by about 60%, from 10.5 to 4.2 (still remarkably high). Figure 3 shows the expected relative risk by distance obtained from the exponential decay with threshold model and the point estimates of Table 3. The estimated effect of AC occupational exposures on MM risk is approximately 3 times that of residential distance exposure at the source.

Spatial clustering of cases was statistically significant according to the T _3_ Cuzick-Edwards test (*p* = 0.003). Second-order distance methods showed evidence of spatial aggregations of cases of average dimension approximately 300 m (Diggle’s *D*-test *p* = 0.016). Similar results were obtained both when the analyses were restricted to non-occupationally exposed subjects and when all subjects were included.

## Discussion

This study has shown substantial effects of environmental asbestos exposure on mesothelioma risk in an Italian town with an important asbestos cement plant. Available data on asbestos fibers concentration in Casale had already suggested relatively high levels of exposure, with average concentration of 48.4 AF/L (total fibers) and of 1.5 AF/L (total amphiboles) in 1991 (Chiappino et al. 1991; Marconi et al. 1989). These measurements, however, are inadequate to describe fiber concentration at a small area scale. Therefore, we have estimated an exposure–response relationship using the distance from the AC factory as a proxy for environmental asbestos exposure. Two key sets of findings are discussed below.

First, there was a substantial risk from residential distance exposure. Residence at the location of the asbestos cement factory had a relative risk for mesothelioma of 10.5 (95% CI, 3.8–50.1), adjusted for occupational and domestic exposures. Risk decreased rapidly with increasing distance from the factory, but at 10-km distance the risk was still 60% of its value at the source. Second, the estimate of AC occupational risk increased remarkably when residential distance exposure was considered, showing that the relative risk from occupational exposures had been underestimated. The relative risk for occupational exposure was 6.0 (95% CI, 2.9–13.0), but this increased to 27.5 (95% CI, 7.8–153.4) when adjusted for residential distance exposure. The reason for such an increase is that when residential distance exposure is not accounted for, AC occupational risk is estimated by comparing occupationally exposed subjects with a reference group still being exposed to high levels of environmental asbestos, although not through occupation. Residential distance exposure was a very strong confounder of occupational exposure in our data.

Domestic and occupational exposures of any relative were comparatively less important risk factors. They were included in the models as potential confounders (Table 5).

The major strengths of this study are the completeness and reliability of information on residential histories of subjects. Since lifelong residential histories of deceased subjects (the majority) were reconstructed by interviewing relatives and could therefore be imprecise, all information was checked against and supplemented by records of town office registries. All addresses were then geocoded blindly with respect to the case–control status of the subject using a GPS. To account for mesothelioma latency, the addresses of the last 20 years before diagnosis for cases or before the interview/ death for controls were disregarded. We examined a number of different criteria for selecting individuals’ residences for spatial analysis, each of which could introduce a different bias. Among all addresses of each subject, we considered the closest to the factory (Magnani et al. 2001), the longest-held, the average coordinates of all dwellings weighted by the duration of residence, and others. We opted for the most specific longest-held residences.

Similarly, the choice of AC occupational exposure as a proxy for all occupational exposures favored specificity over sensitivity. However, in the area under study, other potential sources of occupational exposure to asbestos were scarce (Magnani et al. 1991) and not associated with the distance from the AC plant, causing at worst nondifferential misclassification.

Potential biases that could have affected the study concerned diagnostic criteria reliability, blindness in assessing both case and exposure status, and proportion of nonresponders; the manner in which these were controlled in the design of the study is described elsewhere (Magnani et al. 2001). A limitation of this study is the small numerosity, which is partially compensated by the richness of information on occupational and residential individual histories that are sufficient to provide robust risk estimates.

The choice of a mixed additive–multiplicative model to estimate the excess risk due to environmental exposure was suggested by the *a priori* consideration that such a model ensure that the excess relative risk estimate had the desirable property of tending to 1 at an infinite distance from the source. Including occupational exposure as well as residential distance as an additive term seemed plausible, as both exposures involve the same substance. *A posteriori*, the goodness of fit of the selected model (Equation 1) was better than that of a similar model in which occupational exposure was included as a multiplicative term, as shown by Akaike Information Criterion (AIC; Akaike 1974) of 361 and 365, respectively.

The functional form of risk dependency on the distance from the AC plant (exponential decay with a threshold) was chosen because this model provided the best fit to data. The AIC of a model including a simple exponential decay with the distance was higher. The inclusion of directional effects did not improve model fit. Other functional forms (risk peaked at a given distance from the source) could be considered (Figure 3), but the added complexity was not justified in terms of improved goodness of fit. However, there is still residual variability to be explained. In a previous analysis on residents in Casale, Magnani et al. (2001) observed that residential distance exposure risk remained high even at a considerable distance from the factory. This prompted the hypothesis that sources of exposure other than air pollution from the AC factory could be involved in the increased mesothelioma risk in the area. Among these sources is transportation of raw asbestos and AC materials to and from the railway station and to and from a warehouse. Furthermore, potential sources included the use of AC residuals such as mixing them into the soil to create hard pavement and improve water absorption or applying layers of finely ground AC—rich in asbestos fibers—in the lofts for thermal insulation. The effects of some of these sources were partially considered by adjustment in the models for domestic exposure, which comprised information on the presence of asbestos at home or in the courtyard or garden. Indeed, the results of spatial clustering tests give some support to the hypothesis of secondary sources of asbestos in the area. Further assessments are needed to understand their exact nature, location, and the entity of their effect, although this present study has clearly shown the major role of the AC factory as the principal source of asbestos pollution in the area of Casale.

In conclusion, this study provides strong evidence of an increased mesothelioma risk from asbestos environmental pollution from an industrial source. Although it is generally believed that environmental exposure is a less powerful determinant of MM risk than occupational exposure, this study shows that the strength of the effect of pollution from industrial sources on the general population can reach alarming magnitudes, up to one-third of the risk for asbestos industry workers. Conversely, failing to adjust for environmental exposure may lead to serious underestimation of the relative risk due to occupational exposure in population-based case–control studies carried on in such settings.

## Figures and Tables

**Table 1:** Characteristics of study participants.

Characteristics	Cases *n* (%)	Controls *n* (%)
Sex		
Male	60 (58.3)	166 (61.0)
Female	43 (41.7)	106 (39.0)
Vital status		
Dead	96 (93.1)	251 (92.3)
Alive	7 (6.9)	21 (7.7)
Age (mean ± SD)	65.2 ± 11.9	65.4 ± 11.7

**Table 2:** Risk of malignant mesothelioma of the pleura in Casale in relation to occupation in the AC industry, indoor or outdoor domestic exposure to asbestos (asbestos materials in the garden, courtyard, roof, inside the house), and occupation in the AC industry of any relative.

			Adjusted for age and sex		Adjusted for age, sex, and AC occupation	
	Cases *n* (%)	Controls *n* (%)	OR (95% CI)	AIC	OR (95% CI)	AIC
AC occupation	28 (27.2)	14 (5.2)	7.1 (3.5–14.3)	416		
Domestic exposure	51 (49.5)	98 (36.2)	1.7 (1.1–2.7)	443	1.5 (0.9–2.4)	415
Relatives’ AC occupation	24 (23.3)	22 (8.1)	3.4 (1.8–6.5)	434	2.4 (1.2–4.8)	412

AIC, Akaike Information Criterion.

**Table 3:** Risk of malignant mesothelioma of the pleura in Casale in relation to the distance of each individual’s longest-held residence (after exclusion of 20 years from the date of the diagnosis) from the AC plant.

Distance from the AC plant (km)	Cases *n* (%)	Controls *n* (%)	Adjusted for age and sex OR (95% CI)	Adjusted for age, sex, AC occupation, domestic exposure,^a^ and relatives’ AC occupation^b^ OR (95% CI)
0–3	35 (36.1)	54 (21.6)	7.3 (2.9–18.7)	5.1 (1.9–13.4)
3–5	16 (16.5)	13 (5.2)	14.3 (4.7–43.6)	12.1 (3.9–37.9)
5–7	15 (15.5)	33 (13.2)	5.1 (1.8–14.3)	3.9 (1.3–11.2)
7–9	11 (11.3)	17 (6.8)	7.3 (2.4–22.7)	5.0 (1.5–16.2)
9–11	9 (9.3)	15 (6.0)	6.9 (2.1–22.4)	5.8 (1.7–19.3)
11–13	4 (4.1)	37 (14.8)	1.2 (0.3–4.7)	1.2 (0.3–4.7)
13–15	1 (1.0)	13 (5.2)	0.8 (0.1–7.5)	0.9 (0.1–7.9)
> 15	6 (6.2)	68 (27.2)	1 AIC: 382	1 AIC: 366

AIC, Akaike Information Criterion.

a Indoor or outdoor domestic exposure to asbestos (asbestos materials in the garden, courtyard, roof, inside the house).

b Occupation in the AC industry of any relative.

**Table 4:** Risk of malignant mesothelioma of the pleura in Casale for nonoccupationally exposed residents in relation to the distance of their longest-held residence (after exclusion of 20 years from the date of the diagnosis) from the AC plant.

Distance from the AC plant (km)	Cases [*n* (%)]	Controls [*n* (%)]	Adjusted for age and sex [OR (95% CI)]	Adjusted for age, sex, domestic exposure,^a^ and relatives’ AC occupation^b^ [OR (95% CI)]
0–3	26 (37.1)	45 (19.0)	7.8 (2.8–21.8)	6.8 (2.4–19.5)
3–5	13 (18.6)	13 (5.5)	14.7 (4.4–49.0)	12.5 (3.7–42.2)
5–7	10 (14.3)	31 (13.1)	4.3 (1.3–13.6)	3.9 (1.2–12.6)
7–9	5 (7.1)	16 (6.8)	4.1 (1.1–16.2)	4.2 (1.1–16.5)
9–11	6 (8.6)	15 (6.3)	5.6 (1.5–20.8)	5.6 (1.5–21.1)
11–13	4 (5.7)	36 (15.2)	1.6 (0.4–6.2)	1.6 (0.4–6.5)
13–15	1 (1.4)	13 (5.5)	0.9 (0.1–8.8)	1.0 (0.1–9.9)
> 15	5 (7.1)	68 (28.7)	1 AIC : 310	1 AIC : 307

AIC, Akaike Information Criterion

a Indoor or outdoor domestic exposure to asbestos (asbestos materials in the garden, courtyard, roof, inside the house).

b Occupation in the AC industry of any relative.

**Table 5:** Relative risk (RR) of malignant mesothelioma of the pleura in Casale in relation to occupation in the AC industry, domestic exposure, and occupation in the asbestos cement industry of any relative.

Exposure	Model without distance^c^ [RR (95% CI)]	Model with distance^d^ [RR (95% CI)]
AC occupation	6.0 (2.9–13.0)	27.5 (7.8–153.4)
Domestic exposure^a^	1.3 (0.6–2.7)	1.3 (0.8–2.3)
Relatives’ AC occupation^b^	2.1 (1.0–4.5) AIC: 384	1.4 (0.7–2.9) AIC: 361

AIC, Akaike Information Criterion.

a Indoor or outdoor domestic exposure to asbestos (asbestos materials in the garden, courtyard, roof, inside the house).

b Occupation in the asbestos cement industry of any relative.

c Terms included in the model: age, sex, AC occupation, domestic exposure, relatives’ AC occupation.

d Terms included in the model: age, sex, AC occupation, domestic exposure, relatives’ AC occupation, residential distance.
